# Pharyngeal Microbiota in Pre-COPD and COPD: Associations with Clinical Pattern and Respiratory Infection

**DOI:** 10.3390/biomedicines14010037

**Published:** 2025-12-23

**Authors:** Melissa Ferraris, Chiara Pollicardo, Nicole Colombo, Ludovica Napoli, Federica Dal Molin, Gabriele Nicolini, Giovanni Melioli, Fabio Rapallo, Guido Ferlazzo, Diego Bagnasco, Fulvio Braido

**Affiliations:** 1 Respiratory Disease Unit, Department of Internal Medicine (DiMI), University of Genova, 16132 Genova, Italy; 2 Molecular Microbiology Laboratories, Alliance Medical Ltd., 16129 Genova, Italy; 3 Dipartimento di Scienze Chirurgiche e Diagnostiche Integrate—DISC, University of Genova, 16132 Genova, Italy; 4 Medical Affairs, PIAM-Bruschettini Ltd., 16147 Genova, Italy; 5 Research and Development Unit, Phenomix Ltd., 16125 Genova, Italy; 6 Dipartimento di Economia, Università di Genova, Via Vivaldi 5, 16126 Genova, Italy; 7 Dipartimento di Medicina Sperimentale, University of Genova, 16132 Genova, Italy

**Keywords:** pharyngeal microbiota, microbial diversity, respiratory infections, dysbiosis

## Abstract

**Background/Objectives:** The pharyngeal microbiota plays a critical role in respiratory health by supporting immune modulation, colonization resistance, and metabolic functions. Disruptions in this microbial ecosystem are associated with respiratory diseases; however, standard diagnostics often target individual pathogens, overlooking overall microbial dynamics. This study investigates the composition and diversity of the pharyngeal microbiota in three populations: individuals with pre-COPD (with and without concurrent acute respiratory infection [ARI]) and those with stable COPD. **Methods:** Pharyngeal swabs were analyzed using 16S rDNA sequencing on the Illumina MiSeq platform. Taxonomic and functional profiles were generated with MicrobAT^®^, while microbial diversity was evaluated using the Shannon index and PERMANOVA. Group differences in microbiota composition were assessed via Kruskal–Wallis tests and robust PCA. The sample size was estimated at 8 subjects per group to detect significant differences (α = 0.05, 80% power, SD ≈ 20). **Results:** Twenty-nine swabs were collected: 11 from pre-COPD subjects (PC), 9 from ARI patients receiving antibiotics, and 9 from individuals with stable severe COPD. Microbial diversity was preserved in the PC group (100%) but markedly reduced in ARI (25%) and COPD (15%). Microbiota composition differed significantly across groups (R^2^ = 0.371, *p* = 0.001), particularly at the phylum level. Functional analysis revealed minimal deficits in PC (<10%) but major impairments in ARI (81%) and COPD (56%), indicating reduced microbial functional capacity. **Conclusions:** Broad-spectrum microbial analysis highlights the importance of assessing pharyngeal microbiota beyond traditional pathogen detection, offering potential for innovative diagnostic and therapeutic approaches.

## 1. Introduction

The human microbiota represents a complex ecosystem of microorganisms that plays a crucial role in maintaining host homeostasis and health [1]. Recent advances in culture-independent techniques have enabled more comprehensive characterization of these microbial communities across various body sites [2]. The respiratory tract, particularly the pharyngeal region, hosts diverse microbial communities that contribute to respiratory health through colonization resistance, immune regulation, and metabolic functions [3]. Disturbances in these microbial communities have been associated with various respiratory conditions, though the specific relationships between microbiota alterations and disease states remain incompletely understood [4]. Traditional diagnostic approaches typically focus on identifying specific pathogenic organisms through culture-based methods or targeted molecular panels, potentially overlooking the broader ecological context in which these pathogens emerge [5].

The present study employs comprehensive microbiota analysis of pharyngeal swabs to characterize the complete microbial profiles of three distinct populations: pre-COPD controls, patients with acute respiratory infections, and individuals with chronic respiratory conditions. We hypothesized that microbial diversity might progressively decrease along the spectrum from health to chronic disease, reflecting fundamental alterations in respiratory ecology.

## 2. Materials and Methods

Pharyngeal swabs from PC with and without concomitant respiratory infection and from patient with stable COPD (group E according to GOLD) were collected. The sample size was estimated in order to show marked differences in the pharyngeal microbiota parameter among chronic patients, and acute or stable subjects with an underlying respiratory pre-morbidity condition. Thus, assuming a one-way ANOVA with an alpha level of 0.05, a power of 80%, and a common standard deviation of approximately 20, a minimum of 8 subjects per group was required to detect statistically significant differences. The study conformed to the Declaration of Helsinki. Local ethics committee approval was obtained (HSM-ID 14,214—study N°12-2025—24 March 2025). Informed written consent was obtained from each participant. PC was defined, according to the GOLD 2025 report, as the presence of respiratory symptoms (ex. cough, sputum, dyspnea), radiological impairment (as the presence of emphysema on CT scans) and/or early functional impairment not matching the definition of airflow obstruction (FVC/FEV1 < 0.70) at pulmonary function testing [6]. COPD was assessed according to the definition of disease in the GOLD 2025 document [6]. To minimize the risk of confounding factors, non-acute and chronic selected patients reported no exacerbations in the four weeks prior to the visit or had discontinued antibiotic therapy for at least four weeks before sampling. All patients were taking their usual inhaled therapy (LABA/LAMA or ICS/LABA/LAMA), in accordance with their underlying chronic condition, at the time of sampling. Patients were included regardless of smoking status. The pharyngeal microbiota was considered to be partially—representative of that of the respiratory tract: while the nasopharyngeal microbiota, not analyzed in the study, showed significant differences compared to that of the oropharynx [7], the lower pharynx and laryngeal area do not differ significantly from it; therefore, testing this area represents a more clinically feasible alternative without altering the results obtained from microbiome analysis [8]. The pharyngeal sample was obtained using a sterile synthetic swab (eNAT, Copan, Brescia, Italy) gently but firmly rotated along the posterior wall of the oropharynx and tonsillar pillars, avoiding contact with the tongue, teeth, or cheeks to minimize contamination. Swabs were then stored in the eNAT medium (which rapidly inactivates microbial viability so that the specimen is safe and representative of the original bacterial population) and labelled. Swabs were then stored at −20 °C until processed. Microbial DNA was extracted from a 200 µL volume of the eNAT medium, using a SeePrep32 with STARMag 96 ProPrep Tubes (Seegene Inc., Seoul, Republic of Korea). Magnetic rods automatically transfered the magnetic beads-nucleic acid mixture from one well to another, then processed the cell lysis, nucleic acid adsorption, washing and elution. Purified DNA concentration was measured by a Qubit reader (Thermofisher, Waltham, MA, USA) and stored frozen until used. PCR amplifications of the microbial 16S genes were conducted using the primers provided by the Micro-biota Solution B kit (Enzyme Mix 1 solution containing the enzyme mixture for the PCR target, Arrow Diagnostics, Genova, Italy). PCR products were assayed by the Qubit for DNA concentration; their quality was analyzed using agar electrophoresis on an E-Gel Power Snap Electrophoresis System (Thermofisher, Waltham, MA, USA). PCR products were then purified using magnetic beads (AMPure Beads XP, Beckman Coulter, Inc., Brea, CA, USA); after the purification step, amplicons were indexed using Enzyme Mix 2 solution containing the enzyme mixture for the PCR index, the Amp Mix V3-V6 solution of degenerated oligonucleotides for amplifying hypervariable regions V3-V4-V6 of the bacterial 16S rDNA gene, and the oligonucleotide solution for indexing amplified samples with the PCR target (IndexMix). In addition, at the end of this phase, PCR-index products were purified using magnetic beads as described. Before starting sequencing, a pool of different patients’ samples at the proper concentration was prepared. An Illumina MiSeq system platform (Illumina Inc., San Diego, CA, USA) was used for sequencing. The raw sequencing data were uploaded to a bioinformatic system; taxonomic assignment and bioinformatic analysis were performed using the MicrobAT^®^ software (Microbiota Analysis Tool, ver. 1.3.0.; SmartSeq S.r.l., Novara, Italy). In the first phase of the analysis, reads were cleaned by a dedicated algorithm to remove short, low-quality sequences. Taxonomic assignment was then made by aligning the remaining sequences with the reference database (RDP database release 11-update 5). Only sequences that met reference criteria in the alignment phase were associated by the analysis system to the species taxonomic level (minimum length of the sequence aligning with the reference sequence ≥ 80%, similarity percentage ≥ 97%). The results of the data analysis consisted in the number of reads of the different taxa, sorted as phyla, classes, orders, families, genera and species. To evaluate whether bacteria with special functions were present in the microbiota samples from the three groups, subsets of bacteria were clustered to identify specific function; pathogens, commensals and occasionally commensals were identified accordingly. Other bacterial groups that have the capacity of controlling pathogens at mucosal level (such as bacterial competing with *S. pneumoniae*, with *S. aureus*, with *H. influenzae*, with *P. aeruginosa* and with *Moraxellae*) were described [3,4,5,9,10,11]. In addition, bacteria protecting the respiratory mucosae [3] as well as bacteria involved in the activation of the immune response, both specific for adaptive and innate response, were evaluated [12]. Finally, oral microbes, suggestive of a saliva contamination [13] were also detected. To assess which functions are detectable in the pharyngeal microbiota, different species associated with a given function were enumerated in samples obtained from PC patients without acute respiratory infections, used as a control group compared to ARI and COPD patients. Species associated with each function were then confronted with the same bacterial species under different clinical conditions; a qualitative or quantitative decrease was associated with a functional defect.

## 3. Statistical Analysis

The frequency of the different functional subsets (median value, 15th and 85th percentiles) was calculated based on data from the PC group. The number of reads for each taxon (phyla, classes, orders, genera, families, and species), along with their corresponding percentages, was obtained from the MiSeq NGS output. Alpha-diversity was assessed using the Shannon Index, the Equitability Index and Biodiversity Index, which account, respectively, for the proportional abundance of each taxon using a weighted geometric mean, equitability calculated by dividing the Shannon Index by the maximum diversity of the community, and a representation of the number of bacterial species identified [14]. Beta-diversity was evaluated using the non-parametric Mann–Whitney test. Differences in the absolute composition of phyla were analyzed using the Kruskal–Wallis test. To assess overall differences in relative microbiota composition between groups, a Permutational Multivariate Analysis of Variance (PERMANOVA) was performed using the adonis2 function from the vegan R package (ver. 4.5.2). This analysis was based on Euclidean distances computed from ilr-transformed data, with 999 permutations to determine statistical significance. Finally, to explore group separation and identify key contributing variables, a robust Principal Component Analysis (PCA) was conducted on ilr-transformed data. A biplot was generated to visualize sample distribution along principal components, accounting for potential outliers and deviations from normality.

## 4. Results

A total of 29 patients were enrolled: 11 patients affected by PC without acute conditions (6 females and 5 males; median age 62 years, range 35–77); 9 were ARI (7 females and 2 males, median age 78 years, range 38–94, all on treatment with antibiotic therapy for clinical documented respiratory infection); the COPD group included 9 patients (6 males and 3 females, median age 70 years; range 62–81), all belonging to disease cluster E according to the GOLD document [6].

### 4.1. Biodiversity

**Figure 1:**
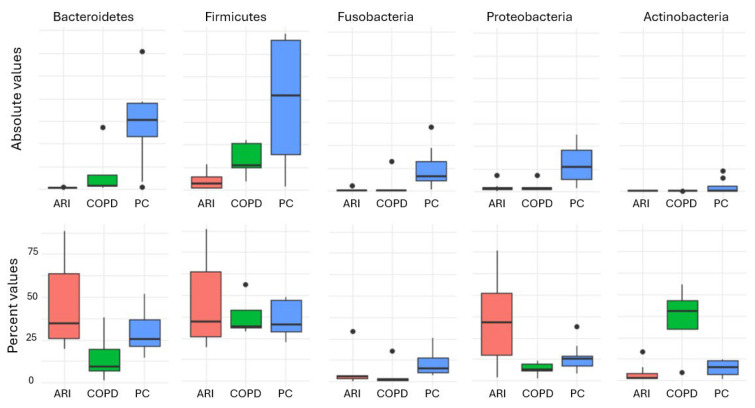
Distribution of the main phyla in the different conditions. In red, ARI patients; in green, PC patients; in blue, COPD patients.

**Figure 2:**
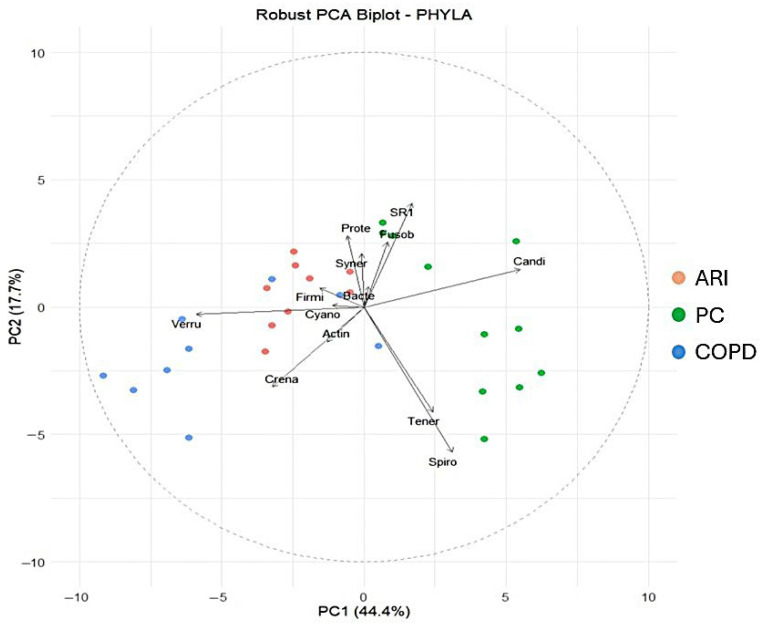
Robust PCA biplot including all detected phyla. In red, ARI patients; in green, PC patients; in blue, COPD patients.

The Shannon diversity index differed significantly among the three groups (H = 17.32, *p* < 0.001). Post hoc analysis using Dunn’s test with Bonferroni correction revealed the following pairwise comparisons: PC vs. COPD: *p* = 0.002; PC vs. ARI: *p* = 0.001; COPD vs. ARI: *p* = 0.015. These findings indicate that microbial diversity was significantly higher in PC compared to both the COPD and ARI groups. The other alpha diversity indexes (i.e., Equitability and Biodiversity) confirmed the results of the Shannon Index. Figure 1 shows the distribution of the main phyla in the different conditions. Statistical analysis of the absolute values (i.e., number of reads, corresponding to the number of bacteria) showed that Phyla were statistically different (Firmicutes *p* = 0.0041; Bacteroidetes *p* = 3 × 10^−4^; Fusobacteria *p* = 2 × 10^−4^; Proteobacteria *p* = 0.0134; Actinobacteria *p* = 0.0021) in the three different conditions (COPD, ARI and PC). Regarding the percent values, statistical analysis (PERMANOVA) revealed a significant effect of the group variable on microbiota composition (R^2^ = 0.371, F = 7.66, *p* = 0.001), indicating that approximately 37.1% of the variance in the ilr-transformed microbial profiles could be attributed to differences between groups. Finally, the robust PCA biplot (Figure 2), calculated on percentage values and including all detected phyla—not just the five previously reported—revealed a clear separation of the PC group along the principal component PC1. This separation was mainly driven by the relative abundance of Candidatus Saccharibacteria, Spirobacteria, and Tenericutes. In contrast, the COPD group was characterized by increased contributions from Verrucomicrobia and Crenarchaeota. The ARI samples displayed an intermediate profile. As expected, core phyla such as Firmicutes and Bacteroidetes were centrally located, reflecting their presence across all groups. Analysis of other taxa (namely, Classes, Orders, Families, Genera and Species) confirmed the results obtained by analyzing Phyla.

### 4.2. Functional Analysis

**Figure 3:**
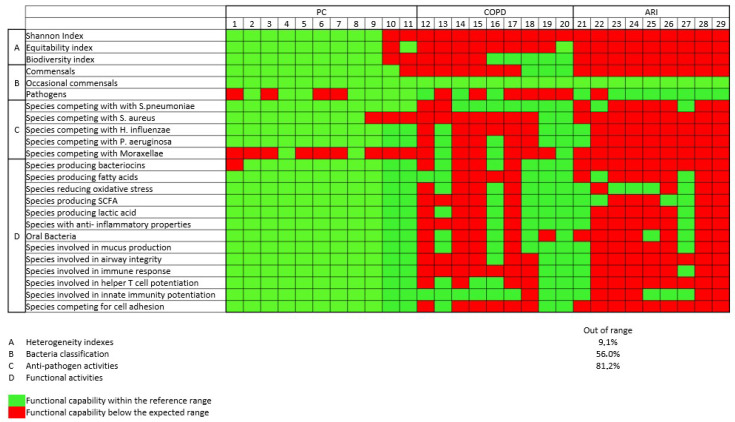
Functional analysis of different functional activities of respiratory tract bacteria. In green, microbial preserved functionality; in red, impaired functionality.

Figure 3 shows the different functional activities of pharyngeal tract bacteria. The frequency of PC with frequency of functional subsets different from the expected was low (<10% of all functions were “depressed”), while in COPD and ARI the frequency was higher (56% and 81%, respectively), indicating a functional defect.

## 5. Discussion

This study demonstrates profound alterations in pharyngeal microbiota diversity and composition associated with respiratory health status. The progressive loss of microbial diversity—from 100% in the PC group to just 15% in COPD, represents one of the most dramatic examples of disease-associated dysbiosis reported in the respiratory microbiome literature. The comprehensive methodology approach enables identification of the complete microbial population, rather than focusing exclusively on standard pathogens typically targeted by conventional culture-based diagnostics or limited molecular panels. This comprehensive perspective supports the view that respiratory infections may be better understood as complex ecological disturbances rather than simple pathogen invasions. The severe depletion of commensal diversity observed in both acute and chronic patients suggests fundamental changes in the respiratory ecological landscape. This finding aligns with the emerging concept that many respiratory infections may represent “dysbiosis-mediated pathogenesis” rather than classic single-pathogen infections [15]. In this model, disruption of the normal microbial community creates ecological niches that facilitate pathogen expansion—potentially explaining the recalcitrant nature of many chronic respiratory conditions, in which antibiotic treatment alone frequently fails to restore respiratory health. As a study focused on identifying evidence of microbiota imbalance in the upper respiratory tract (URT), patients were categorized into broad groups: apparently pre-COPD (PC), with or without an acute infectious episode under antibiotic treatment, and patients with stable severe COPD. Given the limited number of recruited subjects, further sub-grouping was not feasible. Nevertheless, statistically significant differences were observed among the three groups, particularly between PC and the others. Conversely, the latter two groups showed more quantitative than qualitative differences. Being a first application of this methodology to pharyngeal tract microbiota, the sample size calculation was affected by the absence of prior statistical references regarding expected differences. This may have led to potential under- or over-estimation of the studied population. Additionally, considerable heterogeneity was expected due to the nature of pharyngeal microbiota and sampling variability among individuals. Further research is required before pharyngeal microbiota analysis can be applied clinically: for example, although ICS have been shown to reduce bacterial alpha diversity in the respiratory tract, the two most recent studies on the subject have demonstrated this effect only in sputum or alveolar lavage fluid from the lower airways, so it is not yet fully understood whether these drugs may also have a local effect in the pharynx [16,17]; conversely, active cigarette smoking does not appear to significantly modify microbiota stability or the risk of infection due to microbial microenvironment disregulation [18]. Notably, the most substantial differences were evident in the quantitative analysis of URT microbiota. In patients with acute respiratory infections (ARI), the median reduction in bacterial load was −95%, which was expected. Less anticipated was the −80% reduction in patients with severe COPD. This finding may serve as an important biomarker and a possible target for prophylaxis or treatment. Future studies may provide clearer solutions to address this issue. Alpha diversity analysis confirmed a significant reduction in biodiversity in both pathological conditions (COPD and ARI). This was evident not only at the population level (an 80% reduction in PC and 95% in ARI) but also qualitatively, with the emergence of taxa not typically resident in the PC microbiota. These taxa were markedly increased during disease states. As expected, pharyngeal swabs may be contaminated by oral flora, which includes various genera (Table 1). In our samples, oral bacteria accounted for about 20% of microbes in PC and were less represented in COPD and ARI patients. This level of contamination does not appear to significantly affect results. However, higher proportions of oral bacteria should be regarded as a sign of pre-analytical error, likely due to improper sampling technique. Antibiotics have a profound and often non-selective impact on microbiota. While intended to suppress pathogens, antibiotics also cause extensive qualitative and quantitative loss of resident bacteria. In ARI patients, most beneficial functions of commensals are lost. Thus, antibiotics should be used cautiously—not only due to the risk of resistance but also because reducing commensals diminishes nonspecific protective mechanisms, such as pathogen control, mucus production, secretion of protective agents, and production of short-chain fatty acids (SCFAs). URT microbiota imbalance can be attributed to both antibiotic use and chronic respiratory diseases. In the former, cessation of treatment may allow partial restoration of microbial biodiversity. In the latter, it remains unclear whether dysbiosis is a cause or a consequence of disease. In either case, restoring a heterogeneous URT microbiota appears essential. Several strategies have been explored, including the use of gut probiotics based on the gut-lung axis, or specific strains such as Streptococcus salivarius, used in pediatrics to prevent Streptococcus pyogenes infections. Additionally, bacterial lysates (BL)—especially those administered sublingually—seem a promising approach: not only do they reduce respiratory infections but they also positively influence epithelial cells and regulatory systems involving bacteriocins and defensins, contributing to mucosal healing. Alongside probiotics and appropriate dietary interventions, BLs may help restore URT microbiota biodiversity. Our findings support the ecological theory of colonization resistance: diverse microbial communities resist pathogen invasion via niche competition, inhibitory substance production, and immune modulation [19]. The observed collapse in diversity suggests a breakdown of these protective mechanisms. The methodology employed here represents a major advancement over traditional diagnostics by offering context on the entire microbial ecosystem, rather than merely detecting specific pathogens. This ecological lens may help explain why some individuals develop symptoms while others exposed to the same pathogens remain asymptomatic—the outcome may depend on the surrounding microbial environment and community resilience. The relatively small sample size, although supported by a formal statistical framework, may have influenced the final results. This limitation is particularly relevant for poorly represented microbial populations and functional categories. However, future studies with larger cohorts are needed. These should not only refine the enumeration of taxa and the identification of microbes with specific functions, but also incorporate transcriptomic data and, when possible, metabolomic profiling. Such integrated analyses will offer a clearer understanding of the microbial communities of the upper respiratory tract in both health and disease.

## 6. Conclusions

The results of this preliminary study demonstrate that pharyngeal microbiota analysis can distinguish three distinct population profiles: pre-COPD, pre-COPD with concurrent respiratory tract infection, and severe COPD. These profiles show a progressive depletion of microbial diversity. This approach reframes respiratory infections as ecological disturbances rather than simple pathogen invasions, suggesting novel diagnostic applications and therapeutic strategies focused on restoring microbial community structure rather than merely eliminating pathogens. Future studies should investigate longitudinal changes in the pharyngeal microbiota during disease progression along with treatment and recovery to determine whether diversity restoration correlates with clinical improvement. Additionally, interventions aimed at restoring microbiota—such as targeted probiotics or microbiota transplantation—warrant exploration as potential adjunctive therapies for treatment-resistant respiratory conditions characterized by severe dysbiosis.

## Figures and Tables

**Table 1:** Functional activities and relevant bacterial genera.

	Functional Activities	Selected Species from the Following Genera
1	Commensals	Veillonella, Streptococcus, Staphylococcus, Sneathia, Rothia, Prevotella, Neisseria, Micrococcus, Lactobacillus, Kocuria, Haemophilus, Fusobacterium, Corynebacterium, Actinomyces [3,20].
2	Occasional commensals	Propionibacterium, Porphyromonas, Lactococcus, Bacteroides, Actinomyces.
3	Pathogens	Yersinia, Streptococcus, Staphylococcus, Serratia, Rhodococcus, Pseudomonas, Neisseria, Mycoplasma, Moraxella, Legionella, Klebsiella, Haemophilus, Francisella, Escherichia, Enterobacter, Coxiella, Corynebacterium, Clostridium, Chlamydia, Burkholderia, Bordetella, Bacillus, Acinetobacter.
4	Oral microbes	Fusobacteria, Anaerococcus, Catonella, Gemella, Scardovia, Tannerella, Streptococcus, Porphyromonas, Treponema, Fusobacterium, Prevotella, Actinomyces, Veillonella, Aggregatibacter, Campylobacter
5	Competing with S. pneumoniae	Veillonella, Streptococcus, Rothia, Neisseria, Fusobacterium, Dolosigranulum, Corynebacterium [21,22].
6	Competing with S. aureus and H influenzae	Veillonella, Rothia, Neisseria, Lactobacillus, Haemophilus, Dolosigranulum [21].
7	Competing with Pseudomonas aeruginosa	Veillonella, Prevotella, Neisseria, Lactobacillus, Haemophilus, Dolosigranulum, Corynebacterium
8	Competing with Moraxella	Neisseria, Corynebacterium [21].
9	Competing with adhesion sites on cells	Streptococcus, Haemophilus
10	Mucus producers	Lactobacillus, Streptococcus, Corynebacterium, Prevotella, Neisseria, Haemophilus
11	Produce organic acids	Streptococcus, Neisseria, Lactobacillus.
12	SCFA producers	Veillonella, Prevotella, Corynebacterium
13	Lactic acid producers	Streptococcus, Lactobacillus
14	Airway integrity	Prevotella, Neisseria, Haemophilus, Lactobacillus, Streptococcus, Corynebacterium, Veillonella
15	Anti-inflammatory	Veillonella, Streptococcus, Rothia, Lactobacillus, Dolosigranulum, Corynebacterium, Actinomyces, Bifidobacterium, Prevotella, Haemophilus [23].
16	Bacteriocin producers	Streptococcus, Rothia, Neisseria, Lactobacillus, Dolosigranulum, Corynebacterium, Actinomyces
17	Modulation of the immune response	Veillonella, Rothia, Prevotella, Neisseria, Haemophilus, Dolosigranulum, Actinomyces.
18	Innate immunity	Veillonella, Lactobacillus, Dolosigranulum, Corynebacterium, Actinomyces, Streptococcus, Haemophilus, Moraxella.
19	T helper (Th1 and Th17)	Haemophilus
20	Reduction in oxidative stress	Veillonella, Rothia, Prevotella, Neisseria, Lactobacillus, Corynebacterium

## Data Availability

No datasets were generated or analyzed for this study.

## Abbreviations

The following abbreviations are used in this manuscript: PC: Pre-COPD; ARI: Pre-COPD with concomitant acute respiratory infection; COPD: chronic obstructive pulmonary diseases, GOLD: Global Initiative for Obstructive Lung Disease; PCR: polymerase chain reaction; NGS: Next-generation sequencing; IVD: in vitro diagnostic; PCA: robust principal component analysis; URT: upper respiratory tract; SCFA: short-chain fatty acid.
